# An open-label, one-arm, dose-escalation study to evaluate safety and tolerability of extremely low frequency magnetic fields in acute ischemic stroke

**DOI:** 10.1038/s41598-017-12371-x

**Published:** 2017-09-22

**Authors:** Fioravante Capone, Micaela Liberti, Francesca Apollonio, Francesca Camera, Stefania Setti, Ruggero Cadossi, Carlo Cosimo Quattrocchi, Vincenzo Di Lazzaro

**Affiliations:** 1 Unit of Neurology, Neurophysiology, Neurobiology, Department of Medicine, Università Campus Bio-Medico di Roma, Rome, Italy; 2 Fondazione Alberto Sordi - Research Institute for Ageing, Rome, Italy; 3 Department of Information Engineering, Electronics and Telecommunication (DIET), University of Rome “La Sapienza”, Rome, Italy; 4 IGEA Biophysics Laboratory, Carpi, Italy; 5 Diagnostic Imaging, Department of Medicine, Università Campus Bio-Medico di Roma, Rome, Italy

## Abstract

Extremely low frequency magnetic fields (ELF-MF) could be an alternative neuroprotective approach for ischemic stroke because preclinical studies have demonstrated their effects on the mechanisms underlying ischemic damage. The purpose of this open-label, one arm, dose-escalation, exploratory study is to evaluate the safety and tolerability of ELF-MF in patients with acute ischemic stroke. Within 48 hours from the stroke onset, patients started ELF-MF treatment, daily for 5 consecutive days. Clinical follow-up lasted 12 months. Brain MRI was performed before and 1 month after the treatment. The distribution of ELF-MF in the ischemic lesion was estimated by dosimetry. Six patients were stimulated, three for 45 min/day and three for 120 min/day. None of them reported adverse events. Clinical conditions improved in all the patients. Lesion size was reduced in one patient stimulated for 45 minutes and in all the patients stimulated for 120 minutes. Magnetic field intensity within the ischemic lesion was above 1 mT, the minimum value able to trigger a biological effect in preclinical studies. Our pilot study demonstrates that ELF-MF are safe and tolerable in acute stroke patients. A prospective, randomized, placebo-controlled, double-blind study will clarify whether ELF-MFs could represent a potential therapeutic approach.

## Introduction

Ischemic stroke is a leading cause of death and disability worldwide, bearing a deep impact on the socioeconomic burden for healthcare. To date, thrombolysis is the only approved treatment however, the time window is limited to the first hours after stroke, and thus there is an urgent need of different therapies capable to reduce the catastrophic consequences of brain ischemia beyond this short period of intervention. During stroke, acute disruption of blood flow causes pronounced deficit in oxygen and nutrients delivery leading to immediate neuronal death in the core of the ischemic region. Initially, the surrounding tissue, also called the “penumbra”, remains less affected and partially functioning; however, in the following hours, the occurrence of an inflammatory response, with the production of neurotoxic molecules may significantly enlarge the area of neuronal death. Thus, the ischemic penumbra represents the ideal target for neuroprotective strategies aiming to reduce brain ischemic damage^1^.

During the past decades, important advances have been made in better understanding the pathophysiological mechanisms and the signaling pathways involved in the evolution of the ischemic penumbra. Converging evidence suggest that the purine nucleoside adenosine plays a key-role. Adenosine is an important local tissue function regulator, particularly when cellular energy supply fails to meet the demand such occurs in the ischemic conditions^2^. Extracellular adenosine concentrations increase dramatically soon after ischemia and adenosine receptors located both on central nervous system cells and on immune blood cells exert important roles during ischemia and post-ischemic inflammation^2^. Thus, adenosine receptors could be potential targets for neuroprotection and experimental data support this hypothesis. In particular, it has been demonstrated that CGS21680 agonist for the adenosine receptor A_2A_ protects brain tissue in a rat model of transient medial cerebral artery occlusion^3^ by preventing leucocytes infiltration and neuroinflammation following brain ischemia^4^. Even though experimental studies have demonstrated that modulation of adenosine system could be a viable neuroprotective strategy for ischemic stroke, translation to humans has been hampered by the difficulties in producing molecules that selectively activate A_2A_ receptors without inducing desensitization and downregulation and by the occurrence of adverse effects at dosages compatible with a therapeutic effect.

In 2002, for the first time, Varani *et al*. demonstrated the possibility of selectively modulate adenosine receptors using a different strategy, alternative to drugs^5^. Indeed, they showed that a short exposure to extremely low frequency magnetic fields (ELF-MF) caused an upregulation of the A_2A_ receptors expressed by blood cells^5^ and cortical neurons^6^. Subsequently, they demonstrated that the application of ELF-MF has also a direct protective effect on hypoxic damage in neuron-like cells and an anti-inflammatory effect in microglial cells^7^.

The neuroprotective potential of ELF-MF exposure has been also confirmed in animal studies. Grant *et al*.^8^ demonstrated that ELF-MF stimulation attenuated brain damage both on magnetic resonance imaging (MRI) and on histologic examination in a rabbit model of transient focal ischemia. Pena-Philippides *et al*.^9^ confirmed these results in mice showing that such stimulation reduced the infarct size and induced anti-inflammatory and anti-apoptotic effects. Several studies have investigated the effects of ELF-MF in humans demonstrating that such stimulation is safe and can produce measurable effects in healthy volunteers^10,11^ but the effect of this stimulation has been never tested in stroke patients.

Our hypothesis is that ELF-MF could represent an innovative neuroprotective strategy for brain ischemia based on a more physiologic modulation of adenosine receptors because differently from drugs, magnetic stimulation can induce tissue-specific agonist effects without any desensitization and downregulation.

The purpose of this exploratory study is to evaluate the safety and tolerability of ELF-MF in patients with acute ischemic stroke and to collect preliminary data about the interaction of such stimulation with human brain ischemic tissue.

**Figure 1:**
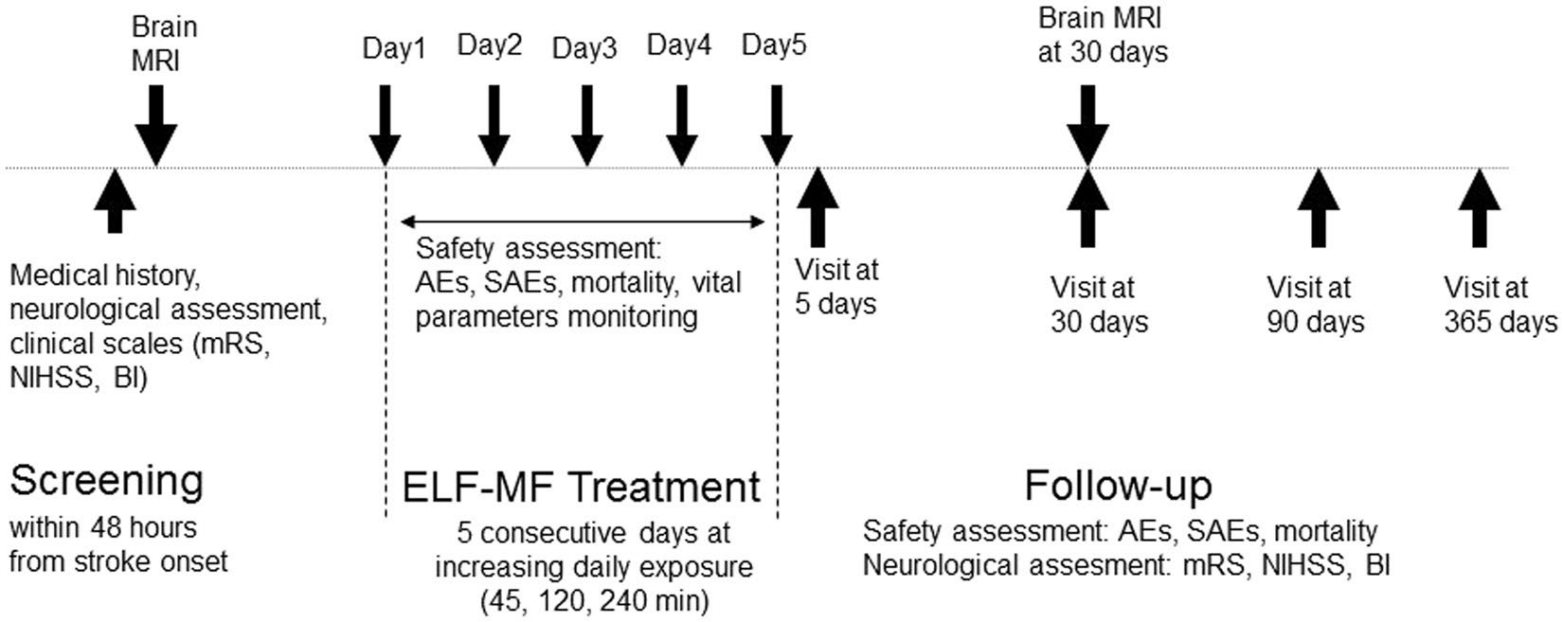
Study design. mRS: modfied Rankin scale. NIHSS: National Institutes of Health Stroke Scale. BI: Barthel Index. AEs: adverse events. SAEs: severe adverse events.

We designed an open label, one arm, dose-escalation, exploratory study. The trial consisted of a 5-days intervention phase and of a 12-months follow-up phase. Within 48 hours from the onset of the stroke, the enrolled patients have undergone clinical evaluation and brain MRI. Subsequently, the patients have undergone ELF-MF treatment, daily for 5 consecutive days. Clinical evaluations were repeated immediately, 30, 90, and 365 days after ELF-MF treatment. Brain MRI was repeated 30 days after ELF-MF treatment (Fig. 1).

## Results

Seven patients (4 M; mean age: 76.3 ± 6.1) were recruited. One patient (F;85) withdrew the consent before the first session of stimulation, thus she was not considered in the analysis. According to the dose-escalation scheme, the first three patients were stimulated for 45 min/day and the following three patients for 120 min/day. Because we did not observe any adverse events (AEs), we propose to the following eligible patients an exposure of 240 min/day but none of them accepted to participate. Indeed, such stimulation was considered too longer by the patients and potentially interfering with the standard of care for acute ischemic stroke by treating physicians. Six patients completed the 5-days treatment period and were included in the analysis. Among these, five patients completed the 12-months follow-up period while one patient was lost at the follow-up after the 3-months visit.

We did not observe any AE both during the treatment and during the follow-up phase. The vital parameters (respiratory rate, heart rate, blood pressure, pulse oximetry, and ECG signal) remained stable during the ELF-MF stimulation. None of the patients required to stop treatment session or reported any discomfort created by ELF-MF stimulation.

Clinical conditions improved in all the patients (Table 1). Such improvement was slightly more evident in the group stimulated for a longer period (120 min). Table 2 shows the MRI volumetric measures of ischemic area, assessed before and after ELF-MF treatment. In the group stimulated for 45 minutes, lesion volume at 1-month follow-up increased in two patients and reduced in one. In all the patients stimulated for 120 minutes, the volume of the ischemic lesion was reduced.

**Table 1:** Clinical features of patients. NIHSS: National Institue of Health Stroke Scale; BI: Barthel Index; mRS: Modified Rankin Scale.

Patients	Stimulation time [min]	NIHSS					BI					mRS				
		Baseline	5 days	1 month	3 months	12 months	Baseline	5 days	1 month	3 months	12 months	Baseline	5 days	1 month	3 months	12 months
1	45	9	7	6	2	2	30	10	25	45	70	4	4	4	4	4
2	45	4	2	0	0	0	55	65	90	100	100	4	4	2	1	0
3	45	5	3	1	1	0	60	65	90	100	100	4	4	2	1	0
4	120	5	3	0	0	0	35	25	100	100	100	3	3	0	0	0
5	120	8	7	6	4	NA	35	35	35	70	NA	4	4	4	3	NA
6	120	6	4	2	1	0	20	30	60	100	100	4	4	2	1	1

**Table 2:** Volumetric and dosimetric measures. DWI Volume PRE is the volume of the ischemic lesion, before treatment, on DWI images; T2 FLAIR Volume POST is the volume of the ischemic lesion, 1 month after treatment, on T2-weighted FLAIR images; Mismatch FLARI-DWI is the difference between the ischemic volume on FLAIR images after treatment and the ischemic volume on DWI images before treatment; FLAIR/DWI rate is the ratio between the ischemic volume on FLAIR images after treatment and the ischemic volume on DWI images before treatment; Dmin - Dmax indicate the distances (minimum and maximum, respectively) of the ischemic lesion from the stimulating coil; Bmin - Bmax indicate the intensity of the magnetic field (minimum and maximum, respectively) induced in the ischemic lesion.

Patient	Stimulation time [min]	DWI Volume PRE [cm3]	T2 FLAIR Volume POST [cm3]	Mismatch FLAIR-DWI	FLAIR/DWI rate	Dmin [cm]	Dmax [cm]	Bmin [mT]	Bmax [mT]
1	45	7,1	27,6	20,5	3,89	3.3	5.5	1.2	1.8
2	45	1,76	1,39	−0,37	0,79	5.7	6.7	1.0	1.2
3	45	11,8	16,0	4,2	1,35	1.6	5.6	1.2	2.2
4	120	25,83	23,15	−2,68	0,90	1.8	6.3	1.0	2.2
5	120	2,51	1,65	−0,86	0,66	2.7	4.5	1.5	1.9
6	120	5,85	3,12	−2,73	0,53	5.1	6.4	1.1	1.3

**Figure 2:**
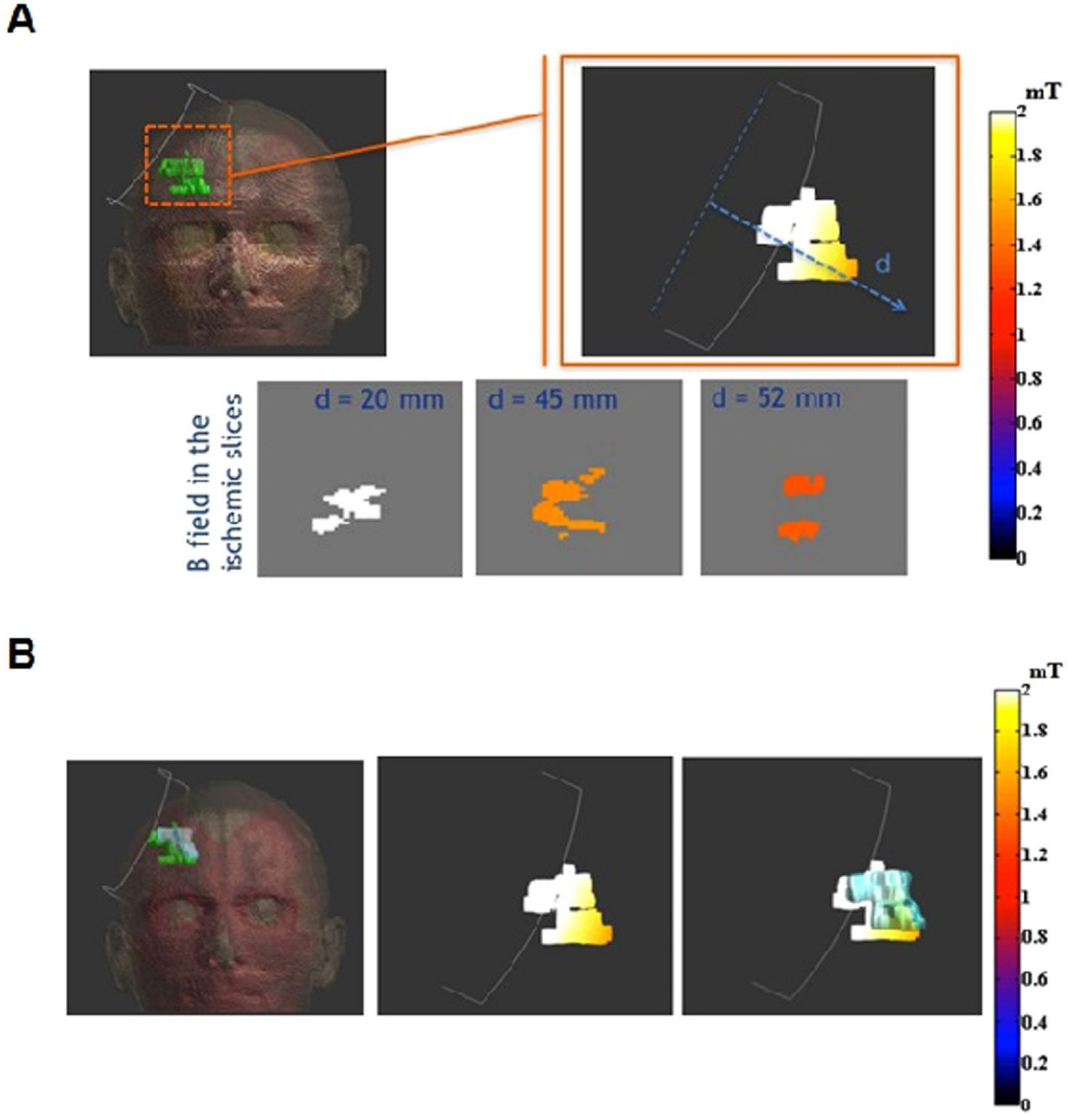
Dosimetric results on exemplificative patient. (**a**) B field in ischemic lesion on the top and on slice perpendicular to the coil axis on the bottom; (**b**) on the left, geometrical comparison between the ischemic volumes before (green area) and after (light blue area) the magnetic field treatment, in the centre, B field on the pre-treatment injured volume; on the right, the same information overlapped with the geometric extension of the post-treatment area.

The distribution of magnetic field in the ischemic lesion and in the surrounding brain tissue was estimated by dosimetry simulation studies. Figure 2 shows the results of a representative patient in the whole head, in the ischemic region and in three slices of the ischemic volume at growing distances from the coil center. For each patient, we have calculated the distances of the ischemic lesion from the stimulating coil and the consequent induced magnetic field range on it (Table 2). The distance ranges from 1.6 cm to 6.7 cm while the intensity of the induced magnetic field in the ischemic lesion ranges from 1.0 to 2.2 mT. Then, the ischemic volume measured after the ELF-MF treatment has been superimposed to the dosimetry results, in order to correlate the volume changing of the injured area to the magnetic field experienced. No correlation was found between the intensity of the induced magnetic field in single ischemic lesions and the evolution of their volume.

## Discussion

To our knowledge, this is the first study that explored the feasibility of ELF-MF stimulation in acute ischemic stroke patients. In a previous study on healthy volunteers, we have demonstrated that ELF-MF do not produce any side effect in humans^11^. In this pilot trial, we confirmed that such stimulation is safe and tolerable also in patients affected by acute stroke.

Indeed, during the 5-days ELF-MF exposure period, vital parameters remained stable and none of the patients experienced neurological worsening (change in NIHSS score), reported any AEs, or required to stop stimulation for any discomfort. These data were confirmed in a long follow-up period (12 months). Indeed, all the patients experienced a progressive improvement in clinical conditions (change in BI and mRS score). The safety of the ELF-MF exposure is also supported by brain MRI that ruled out any hemorrhagic transformation of ischemic lesion. Finally, we also considered a possible effect of ELF-MF on the evolution of ischemic lesion size. The normal evolution of the infarct volume on MRI within first months after stroke is matter of debate^12,13^. In our sample, we observe infarct growth in 2 out of 3 patients treated for 45 minutes per day while in the group stimulated for 120 minutes, all the patients presented a reduction of the ischemic volume. These findings could suggest that ELF-MF exposure of appropriate duration influences the evolution of ischemic lesion toward a reduction of volume infarct.

Dosimetry study shows that, in all patients, the peak intensity of the magnetic field induced in the ischemic lesion was never below 1 mT, the minimum value able to trigger the upregulation of A_2A_ receptors in preclinical studies^5,6^. In addition to the possible effects mediated by adenosine receptors, at these intensities, ELF-MF exposure can exert a direct protective action toward hypoxic insult. Indeed, in neuron-like cells, such stimulation partially restores hypoxia inducible factor-1𝛼 (HIF-1𝛼) activation and inhibits ROS production following hypoxic incubation, while in microglial cells it significantly reduces ROS generation and proinflammatory cytokine release, crucial events in the exacerbation of ischemic condition^7^.

Although intriguing, the neuroprotective effect of ELF-MF needs to be confirmed because the present study was not specifically designed to demonstrate the efficacy of the proposed treatment. Indeed, the small sample size and the lack of a control group do not allow drawing any definite conclusion. On the other side, the use of an exposure system well-validated and already proved effective and safe *in vitro* ^5–7^, in animal models^8,9^ and in healthy volunteers^11^ makes more robust our preliminary findings. The results of dosimetry study showing that the intensity of magnetic field induced in the ischemic lesion is compatible with a biological effect, further support experimental results.

The original design of our study included the recruitment of 9 patients divided in three cohorts with increasing exposure duration: the first 3 patients for 45 min/day, the following 3 patients for 120 min/day and, the last 3 patients for 240 min daily. Planned exposure of 240 min daily was not performed because it was considered unacceptable by the patients and treating physicians. However, the preliminary data obtained in the patients stimulated for 45 and 120 min/day clearly demonstrated the safety and tolerability of the ELF-MF and suggest a possible neuroprotective effect on ischemic lesion. For these reasons, we decided to prematurely stop this pilot trial and planned a prospective, randomized, placebo-controlled, double-blind study to specifically evaluate, in a large sample, the ability of ELF-MF (120 min/day) to promote recovery in acute ischemic stroke patients (NCT02767778). We think that this trial is justified especially given the low-cost and non-invasive nature of the treatment and the promising and converging evidence arising from preclinical studies^5–9^ and the results of the present pilot trial.

In conclusion, our study has demonstrated that ELF-MF stimulation is safe and tolerable in acute stroke patients. The main concerns of this study are the lack of a sham treated group and the small size of the enrolled sample of patients. A prospective, randomized, placebo-controlled, double-blind study will clarify whether ELF-MFs could represent a potential therapeutic approach in acute ischemic stroke.

## Methods

The study complied with the Helsinki declaration and was approved by the local ethics committee (Università Campus Bio-Medico di Roma) and Italian Ministry of Health. Informed consent was obtained from all participants. The study was registered with ClinicalTrials.gov, number NCT01941147 (September 9, 2013). Study protocol has been published^14^.

### Patients

Patient recruitment took place between April 2013 and April 2014 at in-patient unit of the Neurology Department of Policlinico Universitario Campus Bio-Medico, Roma, Italy.

We planned to recruit 9 patients and to treat them according to the following dose-escalation scheme: the first 3 patients for 45 min/day; in the absence of observed adverse events (AEs), the following 3 patients for 120 min/day and, if AEs are still not observed, the last 3 patients for 240 min daily. Because of the exploratory nature of this pilot study, the planned sample size has not been statistically derived.

Eligible patients were >18 years with first mono-hemispheric ischemic stroke, onset of symptoms within 48 hours and National Institutes of Health Stroke Scale (NIHSS) score >4. Exclusion criteria were: acute intracranial hemorrhage; previous ischemic or hemorrhagic stroke; history of seizure; contraindications to magnetic fields exposure (such as implanted metallic parts of implanted electronic devices or other metal in body); life expectancy <3 months; other serious illness or complex disease that may confound treatment assessment; women known to be pregnant, lactating or having a positive or indeterminate pregnancy test; simultaneous participation in another study.

### Procedures

**Figure 3:**
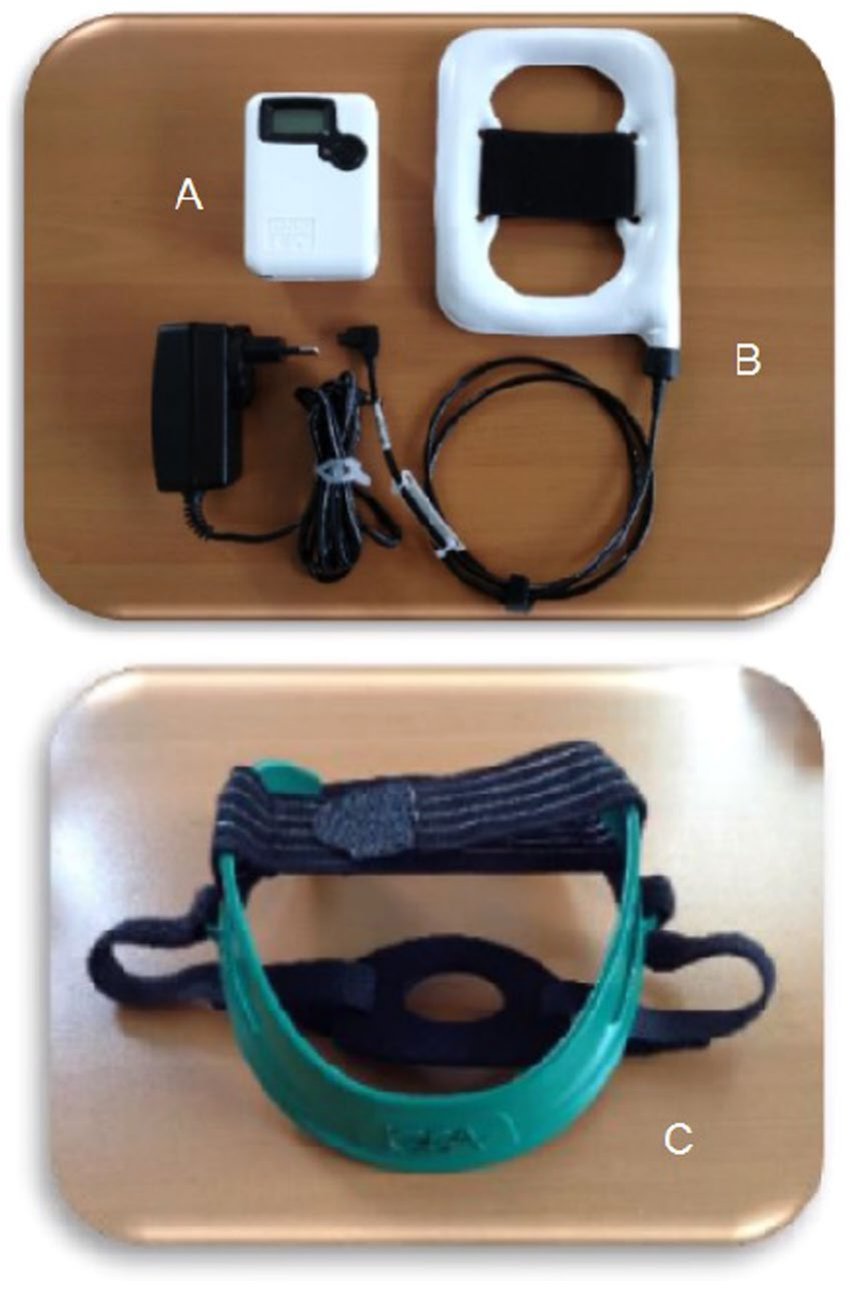
The pulsed ELF-MF stimulation system. Generator (**a**), coil (**b**), and the helmet (**c**) for the positioning of the coil.

The system for delivering pulsed ELF-MF is described in Capone *et al*.^11^. It consists of a custom-made rectangular, flexible coil kept in place by a Velcro strap, upon the ischemic hemisphere. The magnetic pulse generator (B-01; IGEA, Carpi, Italy) supplied the coil with a single-pulsed signal at 75 ± 2 Hz, with a pulse duration of 1.3 ms. The peak intensity of the magnetic field was 1.8 ± 0.2 mT (Fig. 3).

The safety of the ELF-MF was evaluated by measuring the incidence of AEs and mortality throughout the stimulation period and along the 1-year follow-up. Questionnaires on AEs have been administered daily during the whole hospitalization and, after the discharge, at each outward control. Moreover, during the ELF-MF stimulation, patients have been continuously monitored by a multimodal monitor that simultaneously assesses and displays ECG and the relevant vital parameters (respiratory rate, heart rate, blood pressure, pulse oximetry). The tolerability of the stimulation was evaluated by the number of subjects requesting to stop treatment.

Clinical evaluations have been performed by means of international well-validated scales such as NIHSS, Modified Rankin Scale (mRS), and Barthel Index (BI). The scores obtained at follow-up visits were compared with the baseline scores.

All MRI images were obtained with a 1.5 T scanner (Magnetom Symphony, Siemens Medical System, Erlangen, Germany). Sequences were performed in the same order with an initial T1-weighted sagittal localizer: T1-weighted spin-echo sequence in sagittal plane, T2-weighted turbo spin-echo sequence in coronal plane; diffusion-weighted sequence (DWI) in axial plane and T2-weighted Fluid-Attenuated Inversion Recovery (FLAIR) sequence in axial plane. DWI was obtained at three b values magnitude from 0 to 1000 s/mm2. Quantitative measure of volumetric ischemic area region was calculated before treatment on DWI images at high b value (1000 s/mm2) and after treatment on T2-weighted FLAIR images. Image analysis in-house software written in Matlab R2007a (The MathWorks, Inc., Natick, MA, USA) was used for measurement of ischemic lesion volumes. All the lesion areas on a section-by-section basis were segmented with a semiautomated region growing segmentation tool and then manual editing was performed. The semiautomated segmentation tool is based on a simple region-based image method^15^ that involves the selection of initial seed points and following examines neighboring pixels of initial seed point and determines whether the pixel neighbors should be added to the region using a threshold of 20% of seed point signal intensity. An expert neuroradiologist reader blinded to clinical characteristics used this semiautomated technique for initial identification of all the lesions and a manual-editing tool for final corrections to the lesion borders. The number of pixels of segmented abnormal hyperintensity area were automatically summed and multiplied with the slice thickness and the pixel spacing to calculate the volumes of the acute ischemic lesions before treatment and of residual ischemic lesion after treatment. The rate between volumetric ischemic region evaluated on T2 weighted FLAIR images and on DWI scans (FLAIR/DWI) was performed in addition to volumetric mismatch (FLAIR-DWI)^16,17^.

### Dosimetry

The software used for the dosimetric analysis is Sim4Life v.1.2 (ZMT, Zurich MedTech AG), a simulation platform that can deal with medical image data obtained from MRI; in particular, with this software it is possible to convert the typical voxeled data obtained from MRI in volumes and smoothed surfaces and assign electric properties to them. The human head model was obtained from Duke of the ViP (v.1.0, ZMT AG);^18^ we selected a subdomain of 164 × 238 × 178 mm^3^, in order to include all the head structures.

**Figure 4:**
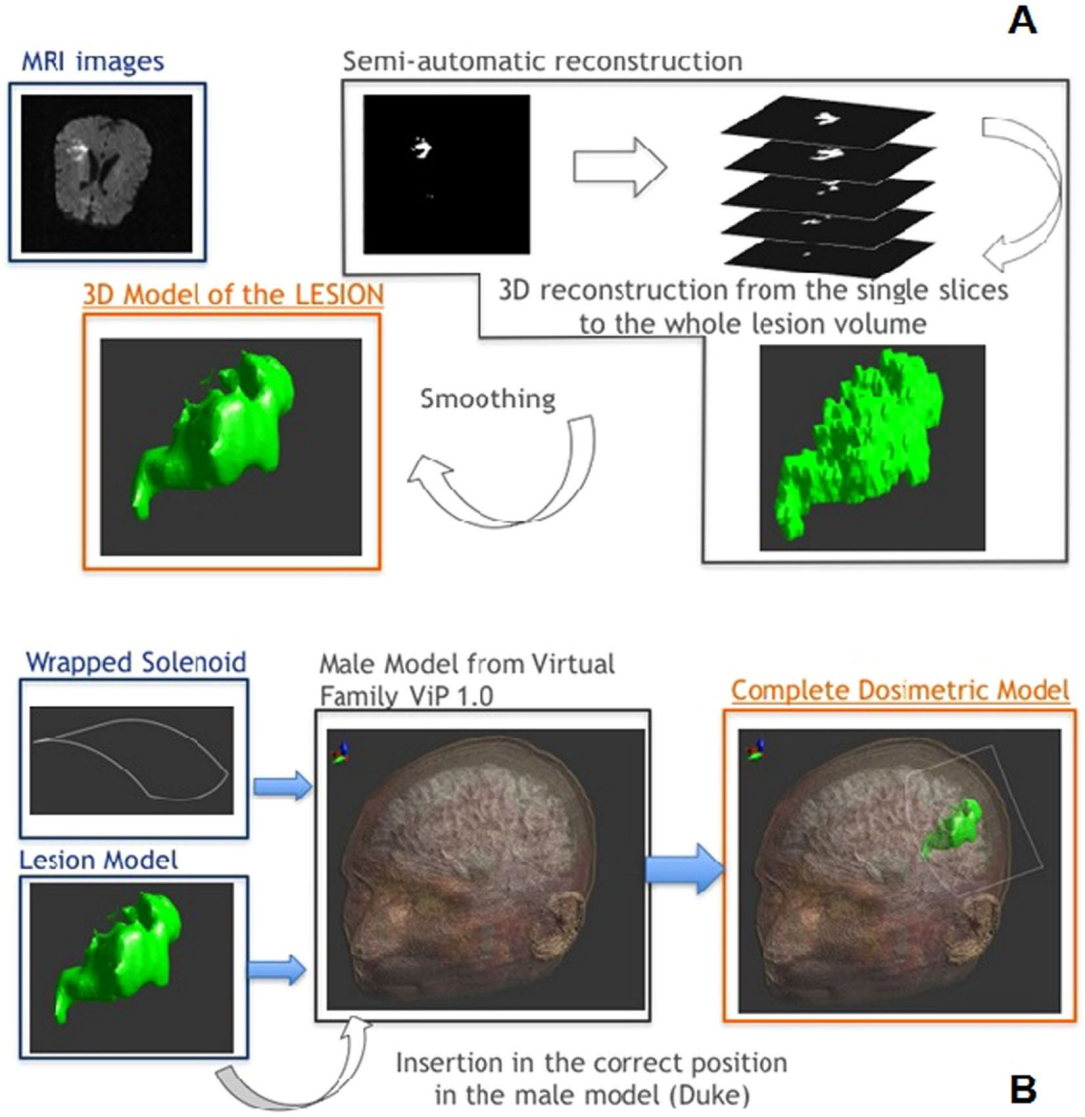
Dosimetric methodology. (**a**) 3D ischemic model obtained starting from MRI data; (**b**) Insertion of the coil model and 3D ischemic model (patient specific) in the human head model.

The geometric model of the ischemic region was obtained directly from MRI images of the patients undergone to the treatment and elaborated with Matlab as previously explained. The ischemic volume was imported in Sim4Life v.1.2 and placed in Duke’s head model; the correct positioning has been achieved looking at the MRI images and estimating the distance of the ischemia from the skull and other brain structures. We assigned to the ischemic tissue the electric properties of the edema^19^, i.e. σ = 1.7 S/m and the same ε_r_ of the gray matter. The coil used in the simulation environment is a single turn rectangular coil of 14 × 10.6 cm; we chose a stimulation current amplitude of 240 A because it is able to generate in the center of the coil a magnetic field intensity of about 2 mT and to reproduce the same magnetic field of the experimental set up. The single turn has been then warped in order to simulate the coil properties of being adaptable to the head shape; this warping has been obtained by computing the mean head curvature in the coronal plane. Then, the coil has been placed as close as possible to the head and in order to have the ischemic volume on the coil axis. Figure 4 shows the final geometry of the model for one patient, the same procedure has been followed for all the patients. Since the presence of the head structures, including ischemia, does not alter the magnetic distribution, all the tissues experience a magnetic field that depends only on the distance from the coil center and the current intensity that feeds the coil. For this reason, the higher is the distance from the coil, the lower is the field intensity.

### Data Availability

All data generated or analysed during this study are included in this published article.
